# Towards automatic quantification of operating table interaction in operating rooms

**DOI:** 10.1007/s11548-025-03363-8

**Published:** 2025-05-04

**Authors:** Rick M. Butler, Anne M. Schouten, Anne C. van der Eijk, Maarten van der Elst, Benno H. W. Hendriks, John J. van den Dobbelsteen

**Affiliations:** 1 Delft University of Technology, Delft, the Netherlands; 2 Leiden University Medical Center, Leiden, the Netherlands; 3 Reinier de Graaf Gasthuis, Delft, the Netherlands; 4 Philips Healthcare, Best, the Netherlands

**Keywords:** Surgical workflow, Workload, Perioperative process, Camera monitoring, Human pose tracking, Robot-assisted surgery

## Abstract

**Purpose:**

Perioperative staff shortages are a problem in hospitals worldwide. Keeping the staff content and motivated is a challenge in the busy hospital setting of today. New operating room technologies aim to increase safety and efficiency. This causes a shift from interaction with patients to interaction with technology. Objectively measuring this shift could aid the design of supportive technological products, or optimal planning for high-tech procedures.

**Methods:**

35 Gynaecological procedures of three different technology levels are recorded: open- (OS), minimally invasive- (MIS) and robot-assisted (RAS) surgery. We annotate interaction between staff and the patient. An algorithm is proposed that detects interaction with the operating table from staff posture and movement. Interaction is expressed as a percentage of total working time.

**Results:**

The proposed algorithm measures operating table interactions of 70.4%, 70.3% and 30.1% during OS, MIS and RAS. Annotations yield patient interaction percentages of 37.6%, 38.3% and 24.6%. Algorithm measurements over time show operating table and patient interaction peaks at anomalous events or workflow phase transitions.

**Conclusions:**

The annotations show less operating table and patient interactions during RAS than OS and MIS. Annotated patient interaction and measured operating table interaction show similar differences between procedures and workflow phases. The visual complexity of operating rooms complicates pose tracking, deteriorating the algorithm input quality. The proposed algorithm shows promise as a component in context-aware event- or workflow phase detection.

## Introduction

Technology plays an increasingly large role in the operating room (OR) [1]. New technologies aim to improve patient safety and procedure efficiency [2]. The adoption of robot-assisted surgery (RAS) has grown in the last few decades. Currently, RAS requires larger teams and more time to perform than minimally invasive surgery (MIS) or open surgery (OS) [3].

RAS, MIS and OS demand different skillsets from surgical staff [4]. Procedures of technical nature shift the focus from direct patient care towards technical activities [5, 6]. This shift impacts work perception and satisfaction of the staff [7].

Added complexity and a shift away from care add stress to an already stressful environment [5–7]. This can diminish quality of care and staff wellbeing. Consequences like communication difficulties, feelings of isolation, and anxiety are quoted. Each of these contributes negatively to patient safety.

Shortages of perioperative staff and high turnover rates are a worldwide concern [7]. Literature identifies workload as a major cause [8]. Beside addressing workload, workflow insights can lead to effective staff deployment and streamlined processes [2].

New technologies should ideally support healthcare professionals without getting in the way or inducing stress. If a technology causes severe changes in workflow, or increases procedure complexity, its design might leave room for improvement. Specifically, some technologies may demand much attention from personnel, thereby shifting focus from direct patient care towards technical tasks.

Knowledge about the effects of technologies on perioperative workflow can aid in the design of new products and support systems. To map these effects, an interesting metric is the time spent on direct patient care. One possible approach to measuring this metric is automatic monitoring of personnel activities in procedure videos. Such monitoring could be deployed in hospitals on a large scale. Outcomes could yield relations between procedure technology levels and perceived workload.

Insights obtained by monitoring from many hospitals could help in the design of future OR technologies. For example, if much time is consistently spent configuring a device during procedures, this reveals an opportunity where user-friendliness can be improved. A new iteration of the product could e.g. carry out the configuration autonomously, or simplify it by making suggestions on its own. This way, the technology assumes a more supportive role, without requiring much attention from the staff. Another application is to optimise planning and logistics for e.g. turnover time and staff wellbeing [9]. Device placement could be updated for better ergonomics or workflow efficiency. Tasks could be divided differently to distribute workload more uniformly over the surgical team.

Computer vision for automated OR monitoring is an upcoming research topic [10]. Bounding box or pose detection can localise individuals in video. Pose trackers infer bodypart—or keypoint—coordinates from all persons in a video on every frame. Detection confidence is scored per keypoint, and each individual is assigned a unique identifier (ID) for re-identification between frames. Most state-of-the-art 2D pose trackers rely on neural networks that need training on annotated images. Some authors provide models that were pretrained on datasets like COCO [11] or MPII [12]. Important to consider is that monitoring itself could introduce discomfort or stress for OR staff. Monitoring systems should be designed carefully and non-intrusively, in a way that does not hinder personnel comfort and wellbeing.

The OR shows visual differences from general-purpose datasets. Clutter, occlusion and visually similar clothing complicate detection and tracking. It cannot be assumed that algorithms trained on general situations perform well in the OR. Reference [13] presents an annotated dataset with recordings of real surgeries. To our knowledge, this is the only such public dataset at the time of writing.

This work presents a first exploration to quantify interaction between staff and the patient from monitoring videos, during procedures of varying technology levels. We take a multimodal approach, where a computer vision algorithm and manual annotations provide complementing measurements of interaction with the operating table and patient. To our knowledge, no automated monitoring tool that measures such perioperative interaction exists in the literature at the time of writing. Patient interaction is annotated based on observed intent and human pose tracklet motion and position are constrained to automatically classify operating table interaction. An interaction metric is designed specifically to counteract bias from missing pose detections.

The “Methods” section describes our dataset, classification of operating table interaction, and experiments. The outcomes are shared in the “Results” section and discussed in the “Discussion” section. Finally, the “Conclusions” section presents our conclusions.

## Methods

### Dataset

**Fig. 1:**
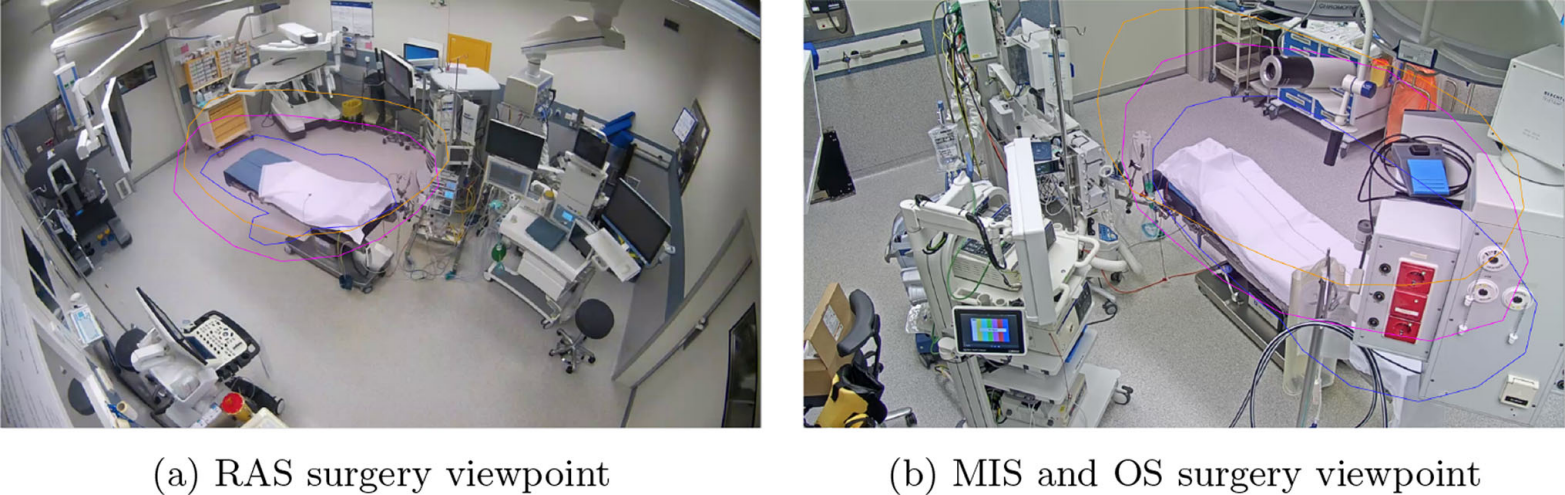
Recording viewpoints in the two ORs. Annotated regions are shown where the wrists (blue), shoulders (purple) and head (orange) must be for a person to interact with the operating table

Videos were recorded in two LUMC ORs during 35 OS, MIS or RAS gynaecological procedures, from the viewpoints shown in Fig. 1. The study was approved by a local medical ethics committee, and all included patients gave informed consent. MIS and OS procedures were filmed using the same synchronised four-camera setup with a resolution of 1920 px $\times$ 1080 px per viewpoint. RAS carried out with the da Vinci surgical platform was filmed with two synchronised cameras with a larger field of view and a resolution of 1280 px $\times$ 720 px.

Each recording was started at anaesthetic induction, and ended after recovery in the OR. In each OR, the camera with the clearest view of the operating table area was selected for interaction quantification. The resulting viewpoints are shown in Fig. 1.

In each procedure, areas were annotated where the wrists, shoulders and head of a person should be present for them to interact with the operating table. Two example annotations are shown in Fig. 1. The wrists area was drawn loosely around the patient in a lying position. Shoulders and head areas were included to correct for the camera 2D projection of 3D scenes.

The personnel activities from the left side of Table 1 were annotated for each person in the room. This was done by two annotators who were unaware of the automatic detection method under development. Annotated activities were grouped into the three categories on the right side of the table, for use in patient interaction classification. Finally, the workflow phases from Table 2 were annotated to enable evaluations per phase.

### Pose tracking

We use AlphaPose [14] to detect poses in the OR. AlphaPose applies a fast human bounding box detector [15, 16], after which features are extracted [17] and a convolutional neural network (CNN) places a pose in each box. During training, a specific loss function and feature normalisation achieve keypoint translation and scale invariance.

A tracker associates detected poses between video frames. AlphaPose includes an optional tracker that uses visual features. This strategy is unsuitable for the OR, as individuals here are dressed similarly. Instead, tracking is done with PoseBYTE [18], which uses only geometric information and prioritises confident detections. PoseBYTE adapts BYTE [19] to associate poses instead of bounding boxes using object keypoint similarity (OKS) [11]. PoseBYTE discards tracklets that are not present for at least two subsequent frames. This compensates for the use of a low-threshold object detector by AlphaPose, which increases the risk of single-frame false positives.

Human bounding boxes are extracted from video with YOLOv3-SPP [15, 16], using features from ResNet152 [17]. A pose is detected in each bounding box using FastPose (DUC) [14] and tracked and refined using PoseBYTE [18]. The pose detector was pretrained by its authors on the COCO dataset [11], and we carried out no further training. PoseBYTE is no machine learning algorithm and therefore requires no training.

**Table 1:**
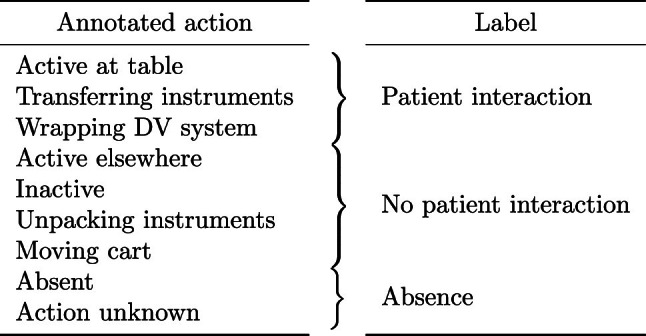
Annotated personnel actions, and their classification as interaction with the patient

**Table 2:**
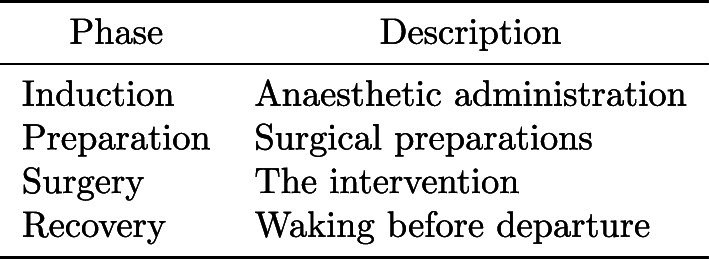
Annotated workflow phases

### Detecting operating table interaction

Our model for detecting personnel interaction with the operating table is visualised in Table 3. When a person is standing still in the correct position, this is assumed to signal interaction with the operating table. These two constraints are detailed in the “Movement” and “Position” sections.

**Table 3:**
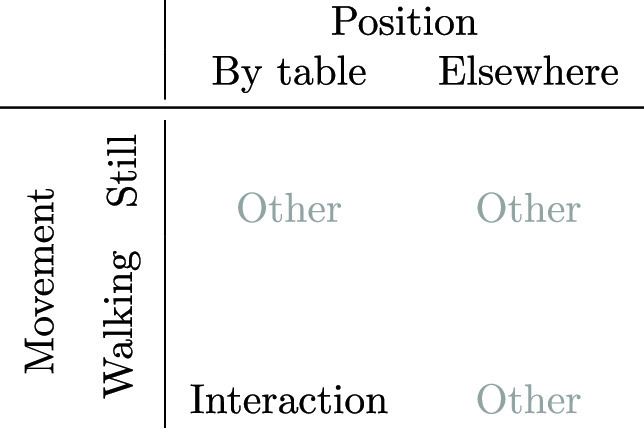
Detecting interaction with the operating table from personnel position and movement

#### Movement

To detect (lack of) movement, we calculate for each keypoint its displacement magnitude in px over a span of $f_\textrm{motion}$ frames. Choosing a larger $f_\textrm{motion}$ enables the capture of longer-term motion. To account for undetected keypoints, each pose is divided into subposes, for each of which movement is classified separately. A subpose $s_\textrm{m}$ is defined to be still if at least a number $M_\textrm{keypoint}^{(s_\textrm{m})}$ of its keypoints $k_\textrm{m}^{(s_\textrm{m})}\subset  s_\textrm{m}$ yields a displacement below a threshold $\tau _\textrm{m}^{(s_\textrm{m})}$. When a keypoint is not detected, or detected with a confidence below a threshold $\gamma _\textrm{m}^{(s_\textrm{m})}$, it is assumed to be still. A pose is defined to be still if at least a number $M_\textrm{subpose}$ of its subposes are.

#### Position

We classify positioning using the annotated regions from the “Dataset” section. Poses are divided into three subposes: (i) the wrists, (ii) the shoulders, and (iii) the head—consisting of the nose, eyes and ears. Each subpose $s_\textrm{p}\in  \{\text {wrists}, \text {shoulders}, \text {head}\}$ is classified to be by the table if at least a number $P_\textrm{keypoint}^{(s_\textrm{p})}$ of its keypoints $k_\textrm{p}^{(s_\textrm{p})}\subset  s_\textrm{p}$ falls within the corresponding annotated region. Keypoints with a detection confidence below a threshold $\gamma _\textrm{p}^{(s_\textrm{p})}$ are not counted within any region. A pose is classified to be by the table if at least a number $P_\textrm{subpose}$ of its subposes is.

### Annotating patient interaction

Detected operating table interaction is intended as a measure for patient interaction. However, it is not guaranteed that operating table interaction as defined in the algorithm indeed signals interaction with the patient. Therefore, the personnel activities from Table 1 were annotated in the dataset in “Dataset” section. These annotations provide a separate measurement of actual interaction with the patient.

### Experiments

#### Models

The used algorithm parameters are shown in Tables 4 and 5. Constraining only a subset of subposes and keypoints compensates for undetected keypoints. Legs are excluded as they are detected least well. As arms can move during operating table interaction, their movement is not considered.

**Table 4:** Values for the parameters defined in the “Movement” section, used to detect pose movement based on two subposes

Parameter	Value	
$f_\textrm{motion}$	5	
$M_\textrm{subpose}$	1	

$s_\textrm{m}$	1	2
$k_\textrm{m}^{(s_\textrm{m})}$	Shoulders	Head
$M_\textrm{keypoint}^{(s_\textrm{m})}$	1	1
$\tau _\textrm{m}^{(s_\textrm{m})}$	17.5 px	17.5 px
$\gamma _\textrm{m}^{(s_\textrm{m})}$	0.3	0.3

**Table 5:** Parameters defined in the “Position” section used to detect pose position

Parameter	Value		
$P_\textrm{subpose}$	2		

$s_\textrm{p}$	Wrists	Shoulders	Head
$k_\textrm{p}^{(s_\textrm{p})}$	Wrists	Shoulders	Nose, eyes, ears
$P_\textrm{keypoint}^{(s_\textrm{p})}$	1	1	2
$\gamma _\textrm{p}^{(s_\textrm{p})}$	0.3	0.3	0.15

#### Classification

We measure the mean time fraction that personnel interacts with the operating table

1 $$\begin{aligned} F&=\frac{1}{\left| P\right| }\sum _{p\in  P}r(p),\end{aligned}$$

2 $$\begin{aligned} r(p)&=\left\{ \begin{array}{ll}1 & \quad \text {If}\,p\,\text {interacts} \\ 0 & \quad \text {Otherwise}\end{array}\right. , \end{aligned}$$

where *P* is the set of all pose detections. Similarly, we measure the mean time fraction of movement by making *r*(*p*) 1 when a pose is moving. The mean time fraction of patient interaction is measured using the annotations, where *P* includes all annotated activities not labelled as ‘absence’.

This definition of *F* compensates for pose tracking errors in several ways. First of all, only detected poses contribute in Eq. (1), i.e. false negatives do not affect *F*. Additionally, summing over all individuals removes any identity-specific information, mitigating re-identification errors. Finally, letting *P* cover a timespan—rather than a single frame—mitigates single-frame detection errors through time averaging.

Equation (1) introduces limitations as well. As detection accuracy varies between workflow phases, *P* will contain more accurate poses during some phases than others. Therefore, if *P* spans multiple workflow phases, this introduces a bias where some workflow phases affect *F* more than others. Another limitation is the equal treatment of all individuals in the room. Discarding information on person roles (e.g. surgeon, nurse, patient, spectator) means that all roles contribute equally to *F*. Patients and spectators therefore affect *F*, whereas our main interest is the interaction of only personnel with the table.

During experiments, we extract *F* for three selections of *P* per procedure type. First, we choose *P* to span all frames of all videos of the same procedure type jointly. The second experiment evaluates *F* per individual video, letting *P* span one video at a time. Finally, we evaluate the evolution of *F* over time within videos. Here, to maintain the time averaging effect, *F* is calculated over a sequence of time windows. Windows were chosen to have a length of 7500 frames, with their start frames spaced 3750 frames apart. Thus, two adjacent windows overlap with ${7500}\,\hbox {frames} - {3750}\,\hbox {frames} = {3750}\,\hbox {frames}$.

Finally, we estimate pose detection performance by evaluating the quantity of detected human poses. The number of pose detections is divided by the number of pose annotations per window. This should yield a value close to 1 if the numbers of annotated and detected poses lie close. Individual pose detections cannot be verified without annotating their location. Note, therefore, that a value of 1 does not guarantee correct pose detections.

#### Qualitative results

Qualitative results are shown with colour-coded pose detections. A pose is drawn  if the person was classified to interact with the operating table. If the person was in the right position, but moving too fast to interact, they are drawn . Finally, a person is drawn  if they were in the wrong position for operating table interaction.

Shown video frames were selected by the authors to demonstrate algorithm successes and failures. Keypoints with a detection confidence below 0.2 were not drawn. For each pose, an ID and a detection confidence score are shown.

## Results

### Dataset

**Fig. 2:**
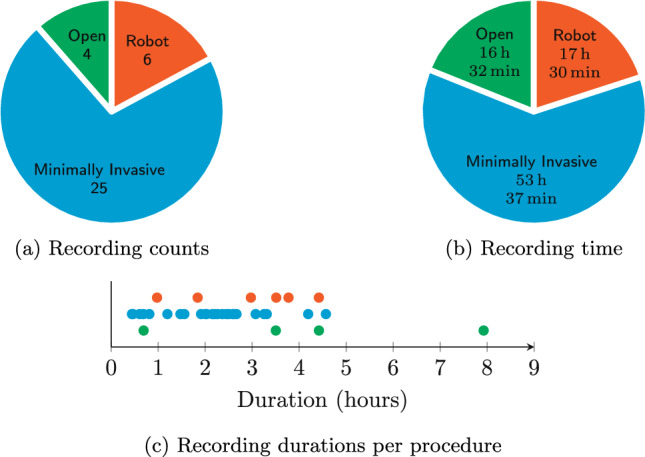
Recorded procedure types and durations

The dataset contains RAS, MIS, and OS procedures in the quantities shown in Fig. 2a. Lighting-dependent framerates range from 6.2 to 26.1 frames per second (fps) during RAS and 12.5 to 25.8 fps during MIS. Since OS is performed with the lights on, the framerate was more constant here: from 24.7 to 25.3 fps. Recording durations are summarised in Fig. 2b, c.

### Operating table interaction over the full dataset

Measured over the entire dataset, the provided algorithm deems personnel to interact with the operating table 30.1% of their time during RAS, 70.3% during MIS and 70.4% during OS. The algorithm classifies personnel as moving 0.8% of the time during RAS, 2.0% during MIS and 2.1% during OS. Annotations report 24.6%, 38.3% and 37.6% patient interaction during RAS, MIS and OS.

### Operating table interaction per video

**Fig. 3:**
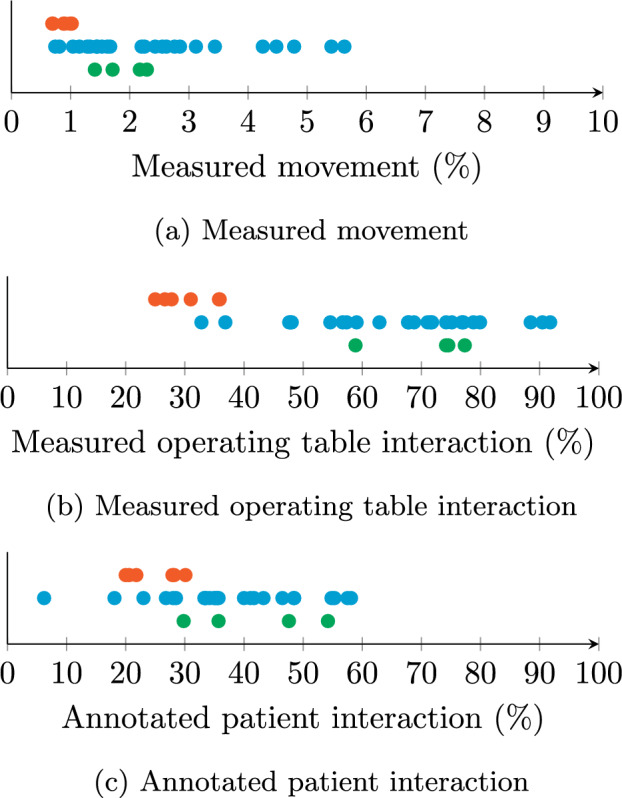
Mean time fractions of movement, measured operating table interaction and annotated patient interaction per video per procedure type

Figure 3a, b shows measured movement and operating table interaction per video. The largest spread is seen between MIS procedures, which range from 0.8 to 5.6% movement and 32.8 to 91.8% operating table interaction time. RAS shows the least movement and operating table interaction time, from 0.7 to 1.0% and 25.0 to 35.9%, respectively. OS has movement between 1.4 and 2.3% and operating table interaction from 58.9 to 77.3%. Annotated patient interactions per video in Fig. 3c range from 20.0 to 30.1% during RAS, 6.2 to 58.1% during MIS and 29.8 to 54.2% during OS.

Two RAS procedures can be seen to have measured operating table interaction time fractions of 35.8% and 35.9%, whereas the rest scores only up to 31%. During one of these, closing the entry wounds took an hour, whereas normally it takes about 15 min. As fewer people are near the table during RAS surgery than wound closure, more operating table interaction is detected during the latter.

Looking at MIS, two procedures show operating table interactions of 32.8% and 36.9%, the others scoring at least 47.4%. During one of these, two spectators are visible and detected during the entire procedure. The personnel at the operating table is poorly visible, due to the patient blanket having the same colour as their clothes. Three other procedures show 88.5%, 90.4% and 91.8% measured operating table interaction. One of the three is a procedure with no spectators and with few people present beside those at the operating table. During another—again without spectators—a complication caused the surgery to take longer than the other workflow phases.

During OS, we observe the opposite as described in the previous paragraph. Here, most interaction with the operating table is observed during surgery. One procedure shows operating table interaction 58.9% of the time, which is at least 74.2% for the others. This procedure has a relatively long anaesthetic induction phase, spanning about one quarter of the recording. As opposed to surgery, few people are around during induction, and preparations are made in parallel throughout the room.

### Interaction over time

**Fig. 4:**
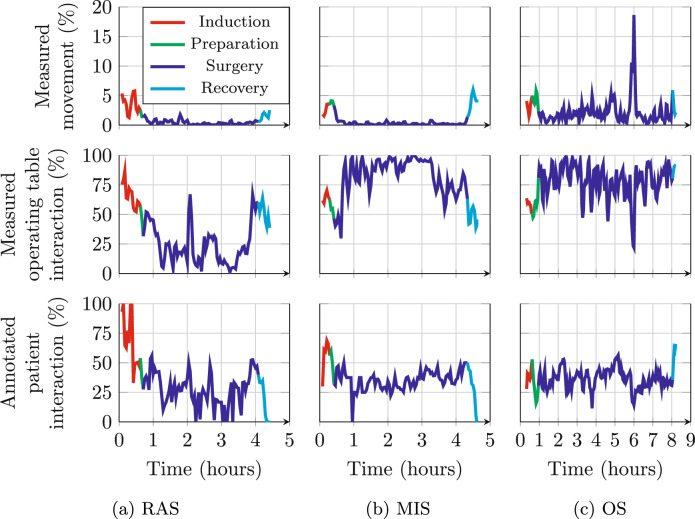
Measured movement and operating table interaction, and annotated patient interaction, over time during an example RAS, MIS, and OS procedure

Figure 4 shows movement, measured operating table interaction, and annotated patient interaction over time during example RAS, MIS and OS procedures. During RAS and MIS, least movement is seen during the surgery phase. MIS and OS show the highest measured operating table interaction during this phase. Annotated patient interaction fluctuates around 40% for all procedures. OS shows most measured variation throughout the procedure.

**Fig. 5:**
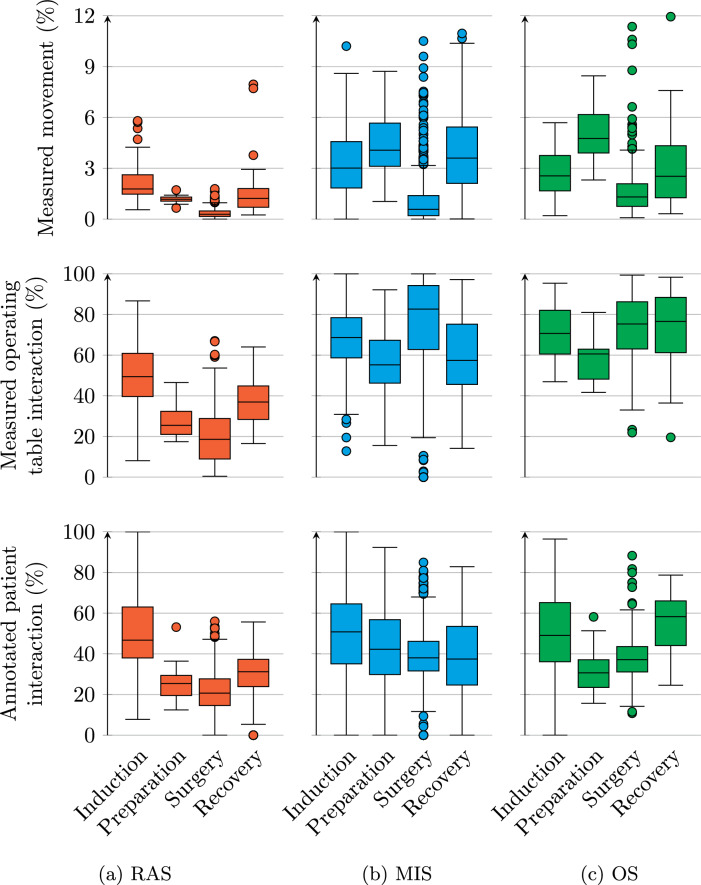
Boxplots of movement, measured operating table interaction, and annotated patient interaction, during different phases and procedure types. Each datapoint is a time window like in Fig. 4. Outliers are present beyond the *y* axis. Note that the range on the *y* axis differs between some subplots. Since datapoints are sampled in time using overlapping windows, measurements are not independent of each other

**Table 6:** Summary of the main results from the “Operating table interaction over the full dataset” to “Interaction over time” sections

	Dataset	Per video	Per window			
			Induction	Preparation	Surgery	Recovery
Robot-assisted surgery						
Table interaction	30.1	$30.3\pm 4.3$	$50.3\pm 15.0$	$28.0\pm 8.2$	$20.4\pm 13.8$	$37.2\pm 11.5$
Movement	0.8	$0.9\pm 0.1$	$2.2\pm 1.1$	$1.2\pm 0.2$	$0.4\pm 0.3$	$1.6\pm 1.5$
Patient interaction	24.6	$24.8\pm 4.1$	$49.6\pm 20.5$	$26.3\pm 9.6$	$21.4\pm 11.4$	$27.1\pm 14.8$
Minimally invasive surgery						
Table interaction	70.3	$66.2\pm 15.4$	$67.3\pm 17.5$	$56.0\pm 16.3$	$76.0\pm 22.9$	$59.6\pm 19.1$
Movement	2.0	$2.5\pm 1.4$	$3.4\pm 2.2$	$4.3\pm 1.8$	$1.5\pm 4.4$	$3.9\pm 2.5$
Patient interaction	38.3	$37.7\pm 12.3$	$47.6\pm 27.0$	$42.2\pm 20.4$	$38.9\pm 14.6$	$37.9\pm 21.9$
Open surgery						
Table interaction	70.4	$71.2\pm 7.2$	$71.9\pm 13.9$	$58.5\pm 10.9$	$73.7\pm 15.5$	$72.4\pm 20.6$
Movement	2.1	$1.9\pm 0.4$	$2.7\pm 1.4$	$5.0\pm 1.5$	$1.7\pm 1.8$	$3.8\pm 4.6$
Patient interaction	37.6	$41.8\pm 9.6$	$50.3\pm 21.9$	$31.9\pm 11.6$	$38.6\pm 11.2$	$55.3\pm 14.9$

Means (%) are reported with one standard deviation (percentage point) where applicable. Note that as RAS was recorded using a different camera system than MIS and OS, these results cannot be compared directly

After 6 h, the OS procedure shows a movement and operating table interaction spike where the surgical team transitions from surgery to closure of the wound. The RAS procedure shows a spike in operating table interaction at 2 h, where a robot arm was replaced. shortly thereafter another increase signals manual repositioning of a robotic arm. The observed spikes are present in the annotated patient interaction during RAS but not during OS.

Measured movement over time per procedure type and phase is summarised in the top row of Fig. 5. Less movement was measured in RAS procedures than other types. During the surgery phase of OS, the peak at 6 h in Fig. 4c shows up as an outlier. Less movement is measured during surgery than during the other phases for all procedure types. During RAS, a lower median movement is measured during preparation than induction, whereas for MIS and OS this is the other way around. median movements during induction and recovery lie at most 0.59% point apart for all procedure types.

The second and third rows of Fig. 5 show measured operating table interaction and annotated patient interaction. Less table and patient interaction are measured and annotated during RAS than other procedure types. The median annotated patient interaction lies higher during induction and recovery than preparation and surgery for RAS and OS procedures. During MIS most operating table interaction is measured during surgery, and least patient interaction is annotated during recovery. The main results from the “Operating table interaction over the full dataset” to “Interaction over time” sections are summarised again in Table 6.

### Detected and annotated poses

**Fig. 6:**
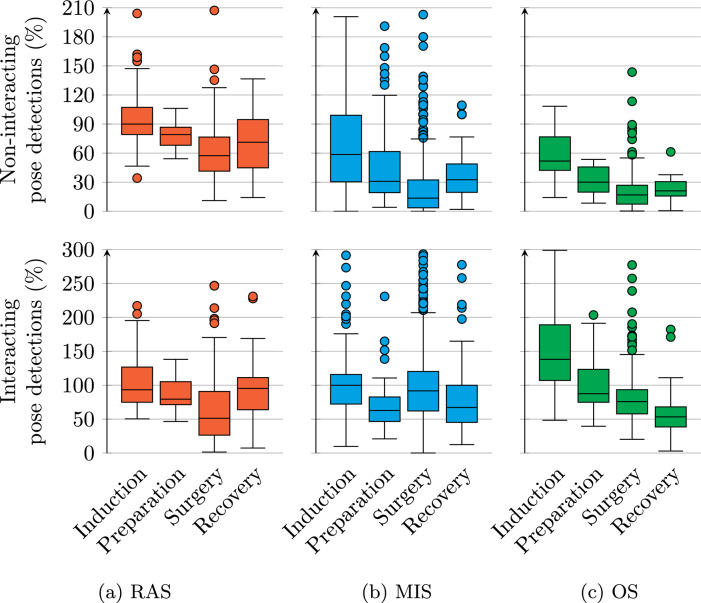
Number of detected poses divided by the number of annotated persons in the room. The calculation was done separately for persons interacting and persons not interacting with the operating table. Each datapoint is a window like in Fig. 4. Outliers are present beyond the *y* axes

Figure 6 summarises the number of detected poses as a percentage of the annotated number of people over time. This was done separately for persons who were annotated and measured as interacting with the operating table, and those who were not. The median number of detected poses is always below 100% for people not interacting with the operating table. For those who interact, detection percentages are higher in most cases. The difference between interacting and non-interacting detection percentages is larger for MIS and OS than RAS. The interquartile spread is also larger for interacting than non-interacting persons.

A larger fraction of non-interacting persons was detected during RAS than during MIS and OS. For interacting persons, detection percentages were more equal between procedure types. The least non-interacting persons were detected during the surgery phase for all procedure types. For interacting persons, this is the case only during RAS. As individual pose detections cannot be verified without annotating person locations, false negatives and false positives might nullify each other in the results of Fig. 6.

### Qualitative results

**Fig. 7:**
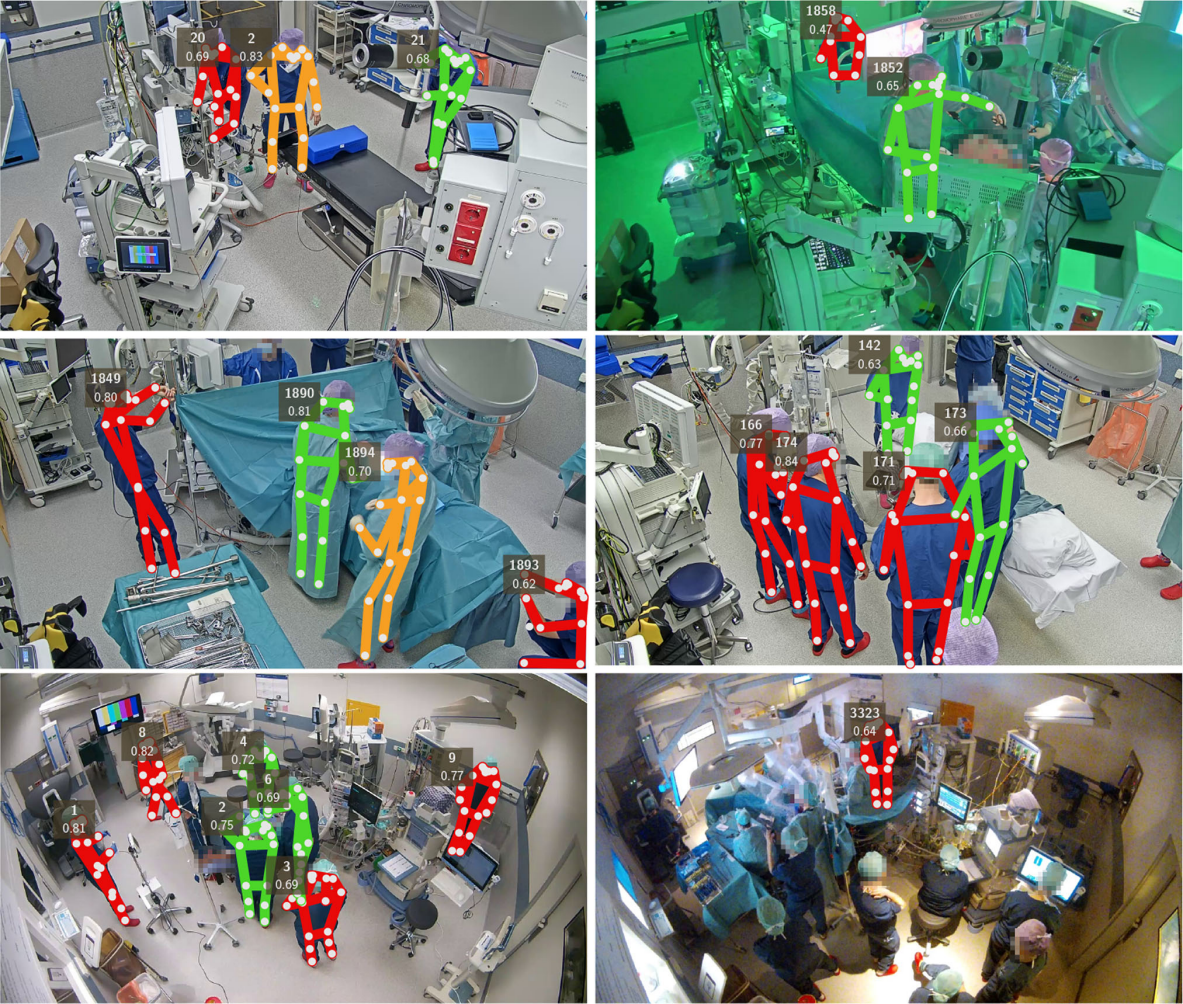
Qualitative pose detections. Poses are drawn in  when classified as interacting with the operating table,  when they are in the right position but moving too fast to interact, and  when they are in the wrong position. A tracking ID and detection confidence are shown for each pose

A sample of human pose detections is shown in Fig. 7. The top-left image shows three correctly detected poses. The cleaning person on the right satisfies the positional and movement requirements, although their activity would not be classified as interacting with the operating table by a human. The person in the middle is not classified as interacting because of their speed. On the left, someone is busy elsewhere, whose left ankle was detected in the wrong place.

The top-right image shows ongoing MIS with the lights turned off. Only two out of five persons are detected. Hips and knees of the person in front are placed despite being occluded. Three staff members near the table are heavily occluded or face away from the camera, and are not detected. The shown IDs of 1852 and 1858 mean that the algorithm assigned and lost IDs 1856 times before this frame.

The middle-left image shows an OS procedure. Persons in front of the camera are detected with confidence scores of at least 0.70, with the exception of the partially out-of-frame person in the lower right corner. The surgeon is classified as interacting with the operating table, one assistant is in the wrong position for this, and another is turning away to move towards the instrument table. Three people in the back are not detected, each of which is either occluded, partially out-of-frame, or both.

The middle-right image was filmed during the induction phase of an OS procedure. Two out of the five detected people are close enough to the operating table to be classified as interacting. Three persons were not detected, all of which are occluded by clothing or another person, or partially out-of-frame.

The larger-field-of-view camera filming the RAS procedures makes persons appear smaller, as can be seen in the fifth image. All persons but one—who is occluded by the IV—are detected with a confidence of at least 0.69. The last image shows a later stage of the same procedure, with the lights off. Only one of the twelve persons is detected here. Looking closely, the sensor noise increased with respect to that when the lights were on.

## Discussion

In this work, we quantified the interaction of personnel with the operating table, by analysing monitoring footage from 35 gynaecological procedures of three technology levels. Personnel movement was measured and interaction with the patient annotated, for a multimodal comparison between workflow phases and procedures of varying technology levels.

Annotated patient interaction suggests less interaction with the patient during RAS than other procedures. This could be caused by the nature of the procedure: personnel is spread through the room during RAS whilst the robot is interacting with the patient and focussed around the operating table and patient during MIS and OS. Measured operating table interaction shows a similar trend, although this result is biased by the differing camera systems and higher-quality pose detections near the operating table. Operating table and patient interaction as a function of time differ similarly between procedure types as well. These differences are more pronounced in measured operating table interaction than the annotated patient interaction. Again, biases from differing camera systems and pose detection quality will amplify measured differences between RAS and the other procedures.

The “Detected and annotated poses” section suggests that a similar percentage of poses is detected during all procedure types. This view could be distorted, as there are more spectators—who are not annotated—during RAS than during MIS and OS. Qualitative results reflect this: since many false negatives here are unannotated spectators, the relative number of pose detections remains high. Detecting spectators reduces measured interaction without affecting the used pose detection metric. Similar reasoning applies to false positives, when persons are detected where there are none. Spectators and false positives explain the detection rates above 100% in “Detected and annotated poses” section.

Lights being off during RAS and MIS causes varying pose detection rates within these procedures. The built-in compensation from the “Classification” section might not be sufficient with false negatives. When comparing results between workflow phases, this needs to be kept in mind.

Least movement is detected during the surgery phase. Here, most persons are busy at the table and spectators are standing still. The other phases show more movement variation, as preparations or cleanup are ongoing throughout the room. Most interaction is detected and annotated during induction or surgery—depending on the procedure type.

The algorithm and annotations measured different kinds of interaction by considering different properties of motion. Patient interaction was annotated based on observed intent and actions, and operating table interaction using only position and displacement of detected poses. Future algorithms could try to capture patient interaction using human action recognition. Here, nuances in intent should be taken into account. For instance, is waiting by the operating table to carry out a task an interaction, or is it idling? Are controlling the robot and monitoring the patient vitals technical or clinical tasks? When looking per procedure or procedure type, patient interaction was lower during RAS and MIS than OS. Within individual procedures, interactions with the operating table and patient evolved differently. For example, during OS, there was interaction with the patient, but not with the operating table, when personnel transitioned from surgery to wound closure.

Large fluctuations are visible in measured operating table interaction, where certain events or workflow phase transitions occur. These events are also visible in the annotated patient interaction, albeit to lesser extent. Hence, the proposed measuring approach might prove valuable for workflow recognition purposes.

This work presents a first step in quantifying time spent on different activities in the OR. In future work, 3D pose detection could be used in the algorithm, which is less dependent on the used camera system [20]. This would mitigate perspective and occlusion issues. A pose detection algorithm should be used that is robust to motion blur and sensor noise in low-light conditions [21]. It should be refined for the OR by e.g. domain generalisation [22] using perioperative monitoring footage like MVOR [13]. A tracking method should be used that corrects for variable framerates. A scalable method, in addition to tracking poses robustly, should not rely on new operating table annotations in each OR. Instead, an object detector could be designed to locate the table automatically. The use of 3D poses solves perspective dependencies, removing the need to annotate or detect separate regions per subpose. The patient interaction annotations in this work could be replaced with a separate classification algorithm. Classifying patient interaction will likely require refined personnel features beyond position and movement, such as roles or action recognition [23].

Recognising the nature of personnel actions can play a role in context-aware systems for improved workflow or staff deployment. The algorithm indicated workflow events and anomalies, which can be used to streamline daily planning and care. For example, the turnover team could be notified when a procedure is finishing. Dashboarding workflow metrics could provide hospitals insight into their operation. This could help reduce expenses and improve workflow through well-informed decision making.

## Conclusions

The presented algorithm is suitable to estimate high-level interaction with the operating table when used with a modern camera system. For lower-level analyses, a more descriptive input feature is necessary that is robust in OR conditions.

## Data Availability

Privacy regulations dictate that the recorded dataset cannot be published. The code is publicly available on https://github.com/RM-8vt13r/Table-Interaction-Detection

## References

[1] Zhang W, Li H, Cui L, Li H, Zhang X, Fang S, Zhang Q (2021) Research progress and development trend of surgical robot and surgical instrument arm. Int J Med Robot Comput Assist Surg 17(5):2309. 10.1002/rcs.2309 10.1002/rcs.2309 34270175

[2] Schouten AM, Flipse SM, Nieuwenhuizen KE, Jansen FW, Eijk AC, Dobbelsteen JJ (2023) Operating room performance optimization metrics: a systematic review. J Med Syst 47(1):19. 10.1007/s10916-023-01912-9 36738376 10.1007/s10916-023-01912-9 PMC9899172

[3] Gillespie BM, Gillespie J, Boorman RJ, Granqvist K, Stranne J, Erichsen-Andersson A (2021) The impact of robotic-assisted surgery on team performance: a systematic mixed studies review. Hum Factors J Hum Factors Ergon Soc 63(8):1352–1379. 10.1177/0018720820928624 10.1177/0018720820928624 32613863

[4] Zheng B, Fung E, Fu B, Panton NM, Swanström LL (2015) Surgical team composition differs between laparascopic and open procedures. Surg Endosc 29(8):2260–2265. 10.1007/s00464-014-3938-3 25361656 10.1007/s00464-014-3938-3

[5] Zamudio J, Woodward J, Kanji FF, Anger JT, Catchpole K, Cohen TN (2023) Demands of surgical teams in robotic-assisted surgery: an assessment of intraoperative workload within different surgical specialties. Am J Surg 226(3):365–370. 10.1016/j.amjsurg.2023.06.010 37330385 10.1016/j.amjsurg.2023.06.010 PMC11234353

[6] Celik SS, Koken ZO, Canda AE, Esen T (2023) Experiences of perioperative nurses with robotic-assisted surgery: a systematic review of qualitative studies. J Robot Surg 17(3):785–795. 10.1007/s11701-022-01511-9 36542241 10.1007/s11701-022-01511-9

[7] Lee SE, MacPhee M, Dahinten VS (2020) Factors related to perioperative nurses’ job satisfaction and intention to leave. Jpn J Nurs Sci 17(1):12263. 10.1111/jjns.12263 10.1111/jjns.12263 31161733

[8] Gil MFH, Hernández JAR, Ibáñez-López FJ, Llor AMS, Valcárcel MDR, Mikla M, Montesinos MJL (2022) Relationship between job satisfaction and workload of nurses in adult inpatient units. Int J Environ Res Public Health 19(18):11701. 10.3390/ijerph191811701 36141970 10.3390/ijerph191811701 PMC9517381

[9] Maier-Hein L, Vedula SS, Speidel S, Navab N, Kikinis R, Park A, Eisenmann M, Feussner H, Forestier G, Giannarou S, Hashizume M, Katic D, Kenngot H, Kranzfelder M, Malpani A, März K, Neumuth T, Padoy N, Pugh C, Schoch N, Stoyanov D, Taylor R, Wagner M, Hager GD (2017) Surgical data science for next-generation interventions. Nat Biomed Eng 1:691–696. 10.1038/s41551-017-0132-7 31015666 10.1038/s41551-017-0132-7

[10] Kennedy-Metz LR, Mascagni P, Torralba A, Dias RD, Perona P, Shah JA, Padoy N, Zenati MA (2021) Computer vision in the operating room: opportunities and caveats. IEEE Trans Med Robot Bionics 3(1):2–10. 10.1109/TMRB.2020.3040002 33644703 10.1109/tmrb.2020.3040002 PMC7908934

[11] Lin T-Y, Patterson G, Ronchi MR, Cui Y, Maire M, Belongie S, Bourdev L, Girshick R, Hays J, Perona P, Ramanan D, Zitnick L, Dollár P (2021) Common objects in context (COCO) . https://cocodataset.org. Accessed 20 June 2023

[12] Andriluka M, Pishchulin L, Gehler P, Schiele B (2014) 2D human pose estimation: new benchmark and state of the art analysis. In: 2014 IEEE conference on computer vision and pattern recognition, pp 3686–3693. IEEE, New York, USA. 10.1109/CVPR.2014.471

[13] Srivastav V, Issenhuth T, Abdolrahim K, Mathelin M, Gangi A, Padoy N (2018) MVOR: a multi-view RGB-D operating room dataset for 2D and 3D human pose estimation. In: MICCAI-LABELS

[14] Fang H-S, Li J, Tang H, Xu C, Zhu H, Xiu Y, Li Y-L, Lu C (2023) AlphaPose: whole-body regional multi-person pose estimation and tracking in real-time. IEEE Trans Pattern Anal Mach Intell 45(6):7157–7173. 10.1109/TPAMI.2022.3222784 10.1109/TPAMI.2022.3222784 37145952

[15] Redmon J, Farhadi A (2018) YOLOv3: an incremental improvement. 10.48550/arXiv.1804.02767

[16] He K, Zhang X, Ren S, Sun J (2015) Spatial pyramid pooling in deep convolutional networks for visual recognition. IEEE Trans Pattern Anal Mach Intell 37(9):1904–1916. 10.1109/TPAMI.2015.2389824 26353135 10.1109/TPAMI.2015.2389824

[17] He K, Zhang X, Ren S, Sun J (2016) Deep residual learning for image recognition. In: Proceedings of the 29th IEEE conference on computer vision and pattern recognition, pp 770–778. IEEE, New York, NY, USA. 10.1109/CVPR.2016.90

[18] Butler RM, Vijfvinkel TS, Frassini E, Riel S, Bachvarov C, Constandse J, Elst M, Dobbelsteen JJ, Hendriks BHW (2025) 2D human pose tracking in the cardiac catheterisation laboratory with BYTE. Med Eng Phys 135:104270. 10.1016/j.medengphy.2024.104270 39922649 10.1016/j.medengphy.2024.104270

[19] Zhang Y, Sun P, Jiang Y, Yu D, Weng F, Yuan Z, Luo P, Liu W, Wang X (2022) ByteTrack: multi-object tracking by associating every detection box. In: European conference on computer vision, pp 1–21. Springer, Cham, Switzerland. 10.1007/978-3-031-20047-2_1

[20] Gerats BGA, Wolterink JM, Broeders IAMJ (2023) 3D human pose estimation in multi-view operating room videos using differentiable camera projections. Comput Methods Biomech Biomed Eng Imaging Vis 11(4):1197–1205. 10.1080/21681163.2022.2155580

[21] Lee S, Rim J, Jeong B, Kim G, Woo B, Lee H, Cho S, Kwak S (2023) Human pose estimation in extremely low-light conditions. In: 2023 IEEE/CVF Conference on computer vision and pattern recognition, pp 704–714. IEEE, New York, USA. 10.1109/CVPR52729.2023.00075

[22] Wang Z, Butler R, Dobbelsteen JJ, Hendriks BHW, Elst M, Dauwels J (2024) Towards robust object detection in unseen catheterization laboratories. In: IEEE international workshop medical measurements and applications, pp 1–6. IEEE, New York, USA. 10.1109/MeMeA60663.2024.10596906

[23] Kaur H, Rani V, Kumar M (2024) Human activity recognition: a comprehensive review. Expert Syst 41(11):13680. 10.1111/exsy.13680

